# Strategies for Bovine Respiratory Disease (BRD) Diagnosis and Prognosis: A Comprehensive Overview

**DOI:** 10.3390/ani14040627

**Published:** 2024-02-16

**Authors:** Mohamed S. Kamel, Josiah Levi Davidson, Mohit S. Verma

**Affiliations:** 1 Department of Agricultural and Biological Engineering, Purdue University, West Lafayette, IN 47907, USA; 2 Department of Medicine and Infectious Diseases, Faculty of Veterinary Medicine, Cairo University, Giza 12211, Egypt; 3 Birck Nanotechnology Center, Purdue University, West Lafayette, IN 47907, USA; 4 Weldon School of Biomedical Engineering, Purdue University, West Lafayette, IN 47907, USA

**Keywords:** BRD, bovine respiratory disease, ultrasonography, blood indicators, prognosis, diagnosis, pneumonia, behavior, markers

## Abstract

**Simple Summary:**

Bovine Respiratory Disease (BRD) poses a significant challenge in managing cattle health. This intricate disease affects both the upper and lower respiratory tracts in cattle and is influenced by various environmental, host, and microbial factors. The lack of standardized definitions complicates diagnosis and treatment, with dairy and beef cattle being commonly affected. Environmental stressors such as overcrowding, transportation, and poor ventilation increase susceptibility, while pathogens like *Mannheimia haemolytica*, *Pasteurella multocida*, *Histophilus somni*, and *Mycoplasma bovis* contribute to its manifestation. Despite antibiotics being the conventional treatment, the rise of antimicrobial resistance is a major concern. Current diagnostic strategies rely on observing clinical signs like fever, cough, and nasal discharge, requiring skilled personnel for accurate detection. However, modern diagnostic tools, including imaging, biomarkers, and automated analysis, offer new perspectives on BRD diagnosis. This review provides a comprehensive exploration of BRD, its existing diagnostic strategies, and the potential of emerging field-based diagnostic technologies for timely and optimal care.

**Abstract:**

Despite significant advances in vaccination strategies and antibiotic therapy, bovine respiratory disease (BRD) continues to be the leading disease affecting the global cattle industry. The etiology of BRD is complex, often involving multiple microbial agents, which lead to intricate interactions between the host immune system and pathogens during various beef production stages. These interactions present environmental, social, and geographical challenges. Accurate diagnosis is essential for effective disease management. Nevertheless, correct identification of BRD cases remains a daunting challenge for animal health technicians in feedlots. In response to current regulations, there is a growing interest in refining clinical diagnoses of BRD to curb the overuse of antimicrobials. This shift marks a pivotal first step toward establishing a structured diagnostic framework for this disease. This review article provides an update on recent developments and future perspectives in clinical diagnostics and prognostic techniques for BRD, assessing their benefits and limitations. The methods discussed include the evaluation of clinical signs and animal behavior, biomarker analysis, molecular diagnostics, ultrasound imaging, and prognostic modeling. While some techniques show promise as standalone diagnostics, it is likely that a multifaceted approach—leveraging a combination of these methods—will yield the most accurate diagnosis of BRD.

## 1. Introduction

Bovine Respiratory Disease (BRD) is a complex condition that arises from a combination of environmental factors, the health status of the host, and microbial interactions [1,2,3]. BRD is estimated to impose an annual financial burden of $800 to $900 million on the U.S. cattle industry, primarily due to calf mortality, medical treatments, and labor costs [4,5]. The multifaceted nature of BRD complicates its diagnosis, often leading to the practice of administering broad-spectrum antimicrobials to suspected cases, thereby promoting antimicrobial resistance within herd subpopulations [6,7,8,9,10]. The term BRD is widely used to describe both upper and lower respiratory illnesses in cattle, which are usually the result of viral and bacterial co-infections. Synonyms for BRD include shipping fever, pneumonia, and undifferentiated fever; however, the lack of a uniform definition can cause confusion in diagnosing and treating the condition. Variations in case definitions across research also hinder the ability to accurately measure the disease’s incidence and prevalence [11,12,13,14]. Clinical manifestations of BRD may be indistinguishable from other diseases, thus, complicating its identification [14,15]. In response to these diagnostic challenges, initiatives are being undertaken to establish precise and consistent definitions based on specific clinical signs and diagnostic criteria. Given its multifactorial nature, BRD requires a comprehensive approach for effective diagnosis and management [16,17,18,19]. Future diagnostic tools should aim to integrate conventional methods, such as laboratory tests, imaging, and automated analysis, into field applications to enhance management strategies. Current diagnostic practices are largely dependent on observing clinical signs by skilled personnel [20,21]. While various methodologies have been developed to evaluate multiple clinical indicators, they still require expert interpretation and often fail to detect all cases of BRD [20,22,23,24,25]. Complementary diagnostic methods such as laboratory testing, imaging, molecular diagnostics, and biomarker tracking can offer additional support but are usually prohibitively expensive [26,27,28,29,30,31,32,33,34,35]. Interestingly, dogs have been shown to discern the scent signatures of various diseases in humans and animals, including BRD. Their olfactory systems can distinguish between healthy and diseased tissues based on volatile organic compound patterns [36,37].

**Figure 1:**
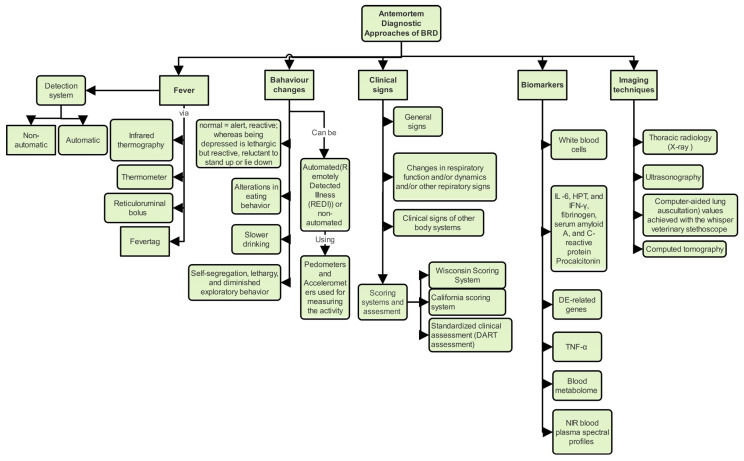
Diagramtic illustration showing the different diagnostic and prognostic approaches of BRD.

This review provides an essential understanding of BRD, its etiology, and conventional treatments. We will review current diagnostic approaches, beginning with clinical and physiological markers assessable in the field by trained personnel (Figure 1). We will delve into imaging techniques used for diagnosing critical cases in clinical settings and explore molecular methods that analyze samples collected on-site without necessitating animal relocation. Finally, we will evaluate emerging technologies aimed at automating diagnostics to deliver superior care in field settings and ensure better outcomes for affected animals.

## 2. Overview of BRD

BRD poses a significant challenge to the cattle industry, impacting both the dairy and beef sectors. The emergence of BRD is driven by an intricate interplay of pathogens, environmental factors, and host-specific characteristics, which complicates its management and prevention—particularly in young calves and during phases of transport [13,38]. Environmental conditions, such as humidity, dust, vehicular exhaust, and temperature variability, are critical in precipitating BRD [39]. Moreover, the stress associated with weaning and transitioning to feedlots intensifies, potentially heightening the incidence of BRD among feedlot cattle [39,40,41]. Routine cattle management practices, including handling and transportation, augment pathogen exposure, thereby elevating the risk of BRD [39]. Calves born to younger dams are especially susceptible to preweaning BRD, with the greatest danger arising post-transport due to contact with pathogens at sales facilities [39,42]. An industry-wide approach that synchronizes incentives and enhances BRD management is vital for effective disease control [39,43]. Implementing proactive measures, such as providing adequate shelter, ensuring proper airflow, offering educational programs, and offering financial rewards can mitigate environmental influences on BRD, consequently lowering healthcare expenses and enhancing productivity.

The complex nature of BRD originates from numerous bacterial pathogens like *Mannheimia haemolytica* (*M. haemolytica*), *Pasteurella multocida*, *Histophilus somni* (*H. somni*), and *Mycoplasma bovis* (*M. bovis*), which thrive under stressors including overcrowding and suboptimal nutrition [44,45,46]. Viral agents, such as bovine respiratory syncytial virus (BRSV), bovine herpesvirus 1 (BHV-1), bovine viral diarrhea virus (BVDV), influenza D virus, bovine rhinitis viruses, bovine coronaviruses, parainfluenza-3 virus, and others, further weaken the immune defenses of calves, paving the way for bacterial infections that lead to serious respiratory issues [13,47,48]. Antibiotics, notably tetracyclines, macrolides, and fluoroquinolones, are frequently utilized to combat BRD, with long-acting formulations providing ease of use in feedlot settings [8,10,49]. However, the prevalent use of antibiotics has given rise to resistant bacteria, highlighting the necessity for prudent use of these drugs alongside all-encompassing management approaches [8,10]. Non-steroidal anti-inflammatory drugs (NSAIDs) are also used to alleviate signs of BRD, with some studies indicating they diminish inflammation and fever [50,51]. Nonetheless, their impact on mortality rates is not well-established [51]. Ongoing research is imperative to ascertain the effectiveness of NSAIDs in enhancing BRD recovery rates [50,51].

## 3. Clinical and Behavioral Tools BRD Diagnosis and Prognosis

### 3.1. Clinical Signs, Clinical Scoring and Clinical Signs Assessment

#### 3.1.1. Overview of Clinical Signs Resulting from BRD

Animals affected with BRD complex display a spectrum of signs, including fever, lethargy, and anorexia [24,52,53,54]. Other manifestations of BRD are coughing, along with nasal and ocular discharges [24,52,53,54]. In severe cases, the disease leads to strenuous, accelerated, open-mouthed breathing [24,52,53,54]. Moreover, BRD has been linked to reduced milk production and inhibited weight gain in affected animals [55]. Calf pneumonia is conventionally recognized as a condition marked by an inducible cough, abnormal respiratory auscultation, a body temperature >39.5 °C, distress, and no involvement of other body systems in fever development [56]. However, there is a lack of consensus in the literature regarding a definitive description for BRD, as well as standardized screening techniques for identifying BRD-positive patients [57,58,59]. These clinical manifestations can last up to five days [60], and depending on the type of pathogen, the onset of signs of infection may take up to nine days. Due to the diverse range of infectious agents responsible for BRD, clinical presentations can significantly differ in intensity and duration [24]. Infections with bacterial pathogens can trigger an acute phase response characterized by a swift emergence of systemic symptoms, such as fever, loss of appetite, lethargy, and respiratory issues including nasal discharge, coughing, rapid breathing (tachypnea), and difficulty breathing (dyspnea) [61]. Additionally, there can be delayed clinical signs of BRD, which may occur with or without fever; typically, neonatal calf diarrhea emerges after considerable damage to the intestinal submucosa [62,63].

#### 3.1.2. Clinical Scoring and Clinical Signs Assessment in BRD Diagnosis and Prognosis

Clinical evaluations are essential for detecting significant indicators of respiratory disorders in animals, highlighting symptoms, such as nasal and ocular discharges, coughing, head tilt, and abnormal or rapid breathing patterns [15]. Veterinarians primarily conduct these assessments as the first step in diagnosing BRD, devising treatments, and managing care themselves, rather than delegating these tasks to veterinary nurses [15]. To bring uniformity to BRD case definitions, multiple scoring systems have been developed and evaluated across various environmental conditions [61,64]. Notably, the Wisconsin Scoring System and the California Scoring System stand out, with the latter including a specialized chart for post-weaning calves and an adapted version [22,23,24,25]. Despite its widespread use, the Wisconsin Scoring System was designed more for treatment guidance rather than as a definitive tool for BRD detection, exhibiting moderate accuracy with 60–80% specificity and sensitivity [22,24,25]. Research conducted in California and Quebec has pinpointed three principal shortcomings of these scoring systems [22,24,25]; a lack of consistency across evaluators due to the identical weight given to each point increase on the 4-scale clinical sign scoring system; the erroneous assumption that each 1-unit increase carries the same risk; and the failure to assign different weights to various clinical signs to reflect their respective importance.

For diagnosing calf respiratory infections, clinical assessments often follow a sequential protocol (Depression, Appetite, Respiration, Temperature, or DART), or they may be conducted in a less structured manner [12,60,65,66]. Unfortunately, the accuracy of these evaluations leans heavily on the experience and judgment of the pen rider, which can introduce a level of subjectivity and hinder the creation of a consensus on case definitions. Studies reviewing these methods have reported inconsistent findings [60]. Moreover, statistical analyses using Cohen’s kappa and Fleiss’ kappa revealed only slight to moderate agreement among raters after training [32]. However, it is believed that more comprehensive training could enhance consistency among evaluators and aid in standardizing disease definitions, despite inherent limitations.

The tenuous link between respiratory symptoms and lung lesions underscores the complexities inherent in the accurate identification of subclinical BRD [67]. The establishment of standardized and dependable diagnostic criteria would facilitate early intervention by enhancing the accuracy of diagnoses [67]. Clinical signs may encompass systemic illness, respiratory distress, and additional signs such as diarrhea or emphysema. In the absence of pronounced outbreaks or a complete spectrum of characteristic signs, the clinical diagnosis of BRD can pose significant challenges.

Scoring consistency was highest using a combination of two scales for assessing discharge, ear position, breathing, and cough [68]. Of these, cough and ear position were the most reliable indicators [68]. The incorporation of rectal temperature measurements with these indicators has the potential to refine the process for straightforward and reproducible screening [68].

The accuracy of clinical sign evaluations could be further enhanced to boost the efficacy of screenings [68]. This enhancement was attempted using video-recorded assessments, which, however, had their own set of constraints, such as fixed viewing angles and the inability to limit the movement of calves. Each clinical sign was analyzed in isolation, thereby mitigating the influence of other symptoms [68]. Pre-awareness of the evaluation criteria by raters may have introduced bias. Additionally, the possibility of rater interactions during simultaneous on-farm assessment could influence individual scores [68]. To surmount these obstacles, clinical scoring systems must undergo continuous improvement through thorough training, meticulous statistical analysis, and rigorously controlled studies aimed at amplifying standardization, reliability, diagnostic accuracy, and a prompt detection of BRD.

### 3.2. Temperature Detection as a Tool for BRD Diagnosis, Prediction, and Prognosis

#### 3.2.1. Fever as a General Non-Specific Sign of BRD and Its Causes

Clinical signs associated with BRD are classified into three groups based on their examination steps: (1) General signs, including depression, behavioral changes, decreased milk production, reduced appetite, and fever; (2) Respiratory function changes, including nasal and ocular discharge, dyspnea, tachypnea, and other respiratory signs like cough and prolonged pharyngeal or oral breathing; and (3) Clinical signs involving other body systems, such as diarrhea and lameness. The consistency of these observed clinical signs across different examinations was documented for both inter-rater and intra-rater concordance.

Fever is a prevalent but non-specific indicator of infectious bronchopneumonia that arises after the initial respiratory pathogen challenge [69]. However, the duration of fever may vary under different conditions and challenges. Despite ongoing infection, fever often resolves rapidly post-tracheal *M. haemolytica* challenge. Detecting fever in sick calves can be challenging due to fluctuations in body temperature [69,70]. Additionally, elevated temperatures may result from non-respiratory or non-infectious factors like heat stress [71]. The accuracy of thermometers and the operator’s technique can also influence rectal temperature measurements [71]. Non-specific symptoms, such as fever, depression, and anorexia, are believed to result from cytokine production and activation by pathogens [69].

#### 3.2.2. Methods for Detecting Fever in BRD

Automated methodologies enable continuous monitoring of cattle body temperature, providing valuable insights into animal health. Researchers utilizing a bolus to measure internal temperatures in 24 animals detected signals of respiratory distress, such as unusual rumen temperature spikes, up to five days before they became apparent [72]. They also established an inverse correlation between elevated rumen temperatures and the average daily gain (ADG) in livestock [72]. This technique has been praised for its high reliability and consistency across different operators [22], although its accuracy may be influenced by variables like distance and weather conditions [73], highlighting the need for method standardization [73]. Despite fever being a potential indicator of BRD, relying solely on this parameter has proven to have a low specificity for accurate diagnosis, as evidenced by suboptimal ultrasound readings in the chest and the absence of clinical signs. The reported study in question may have been limited by its sample size, which could have been too small to yield statistically significant results or to substantiate the conclusions. Moreover, it did not adequately consider the effects of various treatment approaches on lung pathology [74].

The literature advocates for the use of an automated infrared thermography (IRT) system, akin to the one developed by Schaefer et al. which facilitates the automatic detection of BRD in beef calves. This system captures thermal images during calves’ automated feeding sessions [75,76]. In laboratory conditions, IRT has demonstrated remarkable accuracy in identifying increased eye temperatures in beef calves up to four to six days before the emergence of observable BRD signs [77]. When used at a drinking station to monitor eye temperature, IRT can accurately identify clinical BRD cases with 100% sensitivity and 97% specificity on the day of diagnosis. This finding is based on comparisons with parameters such as a core temperature above 40 °C, a clinical score over 3, and white blood cell/neutrophil-lymphocyte ratios below 0.1 or above 0.8 for leucopenia or neutrophilia, respectively [75,78].

#### 3.2.3. Fever as a Method for Detecting and Diagnosing Clinical and Subclinical BRD

Detecting fever is a crucial aspect of diagnosing calves with subclinical BRD. Previous research has delved into the impact of initiating early treatment on BRD progression, with fever serving as a key indicator for treatment decisions [16]. These studies have established fever as a sensitive marker for detecting the disease. However, attributing BRD as the definitive cause of fever remains challenging due to the frequent absence of clinical signs [74]. The association of lung pathology at slaughter and incidents of reticulorumen hyperthermia has been established.

Furthermore, a documented correlation exists between lung pathology at the time of slaughter and episodes of reticulorumen hyperthermia. The reticulorumen bolus method has shown promise as a diagnostic tool for identifying subclinical BRD, as indicated by pulmonary abnormalities discovered during postmortem examinations. While treatment strategies and management protocols are crucial for handling subclinical BRD, further research is imperative. The current literature lacks evidence to confirm the effectiveness of this diagnostic approach or the associated treatment protocols [79].

### 3.3. Behavioral Changes in BRD Diagnosis and Prognosis

Behavioral monitoring plays a pivotal role in evaluating health status [80]. However, the diagnostic sensitivity and specificity for BRD vary widely and are generally low despite integrating multiple indicators, such as visual observation of clinical signs and rectal temperature measurements. On average, 14.4% of cattle in feedlots are diagnosed with BRD; yet, the prevalence within individual pens can range dramatically from 0% to 100%. This wide range may decrease both the likelihood of contracting BRD and the promptness of its detection. Predictive modeling and technological advancements, including refined case definitions, improved diagnostic tests, and targeted antibiotic use, have the potential to enhance BRD management practices [81,82]. Behavioral scoring methods have been shown to have higher sensitivity, but lower specificity compared to pulmonary auscultation. [83]. When compared with ultrasonography, these behavioral scores are less sensitive and specific [84]. Additionally, observing feeding behaviors could aid in the early detection of BRD risk, thus, reducing the treatment duration [85]. Clinical signs of BRD can vary among observers and are often transient [20]. The prevalence of subclinical BRD—pulmonary consolidations without visible signs—can reach as high as 67%, indicating that many calves may be misclassified as healthy [86]. Conventional diagnostic approaches, such as visual observation, auscultation, and clinical assessments, have limitations that can result in misinterpreting associated behavioral changes.

In the absence of systematic on-farm scoring, behavioral assessments serve as an effective substitute for detecting BRD and supporting the refinement of diagnostic protocols [87]. Although research has documented certain visual behavioral changes associated with BRD, the behavior of calves with subclinical BRD remains largely unexplored [87]. The Wisconsin Calf Health Scoring App (https://www.vetmed.wisc.edu/fapm/svm-dairy-apps/calf-health-scorer-chs/ (accessed on 5 February 2024)) offers an attitude score that proves invaluable for early identification of BRD-affected animals, thereby facilitating prompt disease management. Currently, behavioral tests for BRD detection exhibit a sensitivity range of 23–68% and a specificity range of 43–95% [87,88,89]. This variability indicates that behavioral tests alone are insufficient for an accurate BRD diagnosis. This limitation is especially clear when compared with alternative diagnostic methods like calf lung ultrasound, which boasts a high specificity of 94% and sensitivity of 79% [90].

#### 3.3.1. Alertness as a Behavior Change in BRD Diagnosis

Alertness, or its lack thereof, serves as a critical indicator of behavioral alterations linked to illness [91]. Recognized symptoms such as lethargy and diminished exploratory behavior have been incorporated into scoring systems for BRD [92]. Studies comparing calves with clinical BRD, subclinical BRD, or no BRD reveal that those with subclinical conditions do not exhibit significant differences in behavioral scores when compared to healthy calves, indicating that subclinical BRD may not affect alertness or result in depressed behavior [89,93]. In contrast, calves exhibiting clinical signs of BRD demonstrate a decreased propensity to approach humans or new objects relative to their healthy counterparts, suggesting a decline in both alertness and exploratory behavior [88]. Other signs of disease-related changes in alertness include an increased preference for self-isolation and choosing remote locations among adult dairy cows [94]. Automated methods are essential for objectively measuring alertness and other health and welfare indicators in cattle [80,95]. Furthermore, investigating postural changes in preweaning calves could shed light on more nuanced shifts in alertness.

#### 3.3.2. Energy and Feeding, Drinking Behavior, and Other Behavior Changes during BRD

Cattle affected with BRD display changes in behavior and feeding patterns up to four days before visible symptoms appear [96,97]. Proactive measures, such as isolating affected animals, conducting tests, and administering early treatment, can enhance health outcomes and curtail healthcare expenses. Research using a fractional slope linear regression model revealed fluctuations in the feeding rates of growing bulls preceding BRD onset. [98]. Notably, an uptick in feed intake was noted before clinical symptoms became apparent in bulls.

Calves with BRD often exhibit reduced activity, likely a strategy to conserve energy essential for an effective immune response. This “sickness behavior” correlates with a lower appetite and decreased drinking rates, leading to diminished daily gains [91,99]. The underlying cause is the need to save energy for immune functions and the impact of fever and inflammation due to infection [91]. BRD-affected calves are less than their healthy counterparts [100]. Increased energy levels in these animals due to hypermetabolism cause behavioral alterations, including more sleep, less social interaction, and reduced sexual activity and feeding. However, the absence of distinctive behaviors in some acidotic animals can lead to misclassification as BRD [101,102], complicating behavioral assessments [89,103]. The primary distinct feeding trait among the BRD categories was drinking speed; calves with clinical BRD drank more slowly, reflecting findings by Knauer et al. which showed that clinically sick calves were slower drinkers on diagnosis day [104]. Moreover, the drinking rates of clinically BRD calves were similar to those without BRD or with subclinical BRD, indicating some normal calves might have recovered from a previous BRD episode [99]. Eating behavior due to pre-enrollment BRD was not sustained throughout the study period as no feeding behavior differences were observed post-diagnosis between BRD calves and controls. Nevertheless, potential selection bias existed since only surviving calves older than 21 days were included [99].

Wolfger et al.’s discrete survival analysis linked increases in dry matter intake per meal, meal frequency, and meal intervals with reduced BRD development risk up to a week before clinical signs emerged [85]. This supports previous findings that pattern recognition can identify significant feeding behavior deviations in cattle before obvious BRD signs occur [105]. Lukas et al. also reported a decrease in milk yield in cows with mastitis and reduced water intake due to fever [106]. Beyond feeding habits, other behaviors have been explored for BRD diagnosis utility. Dairy calves with BRD showed multiple abnormal clinical signs and a lower tendency to approach new objects or stationery persons on diagnosis day [88]. An evaluation encompassing five behaviors—abnormal lying or standing posture, lethargy, group isolation, and two approachability tests—can be employed on farms where automatic feeders are not available [87]. Precise technology devices have shown that preweaning dairy calves with BRD exhibit decreased milk and starter intake, increased lying times, fewer lying bouts, lower step counts, and a diminished activity index compared to healthy calves [95,107].

#### 3.3.3. Lameness as a Behavior Change in BRD Diagnosis

Behavioral changes stemming from lameness may play a role in the onset of BRD, as affected animals are prone to isolating themselves and moving to less optimal areas of the barn. Moreover, a direct connection between BRD and lameness could exist due to the impact of *M. bovis* on both lungs and joints, with arthritis-like symptoms often manifesting one to two weeks post-infection [108,109]. Notably, initial observations indicated that when BRD was present alongside lameness as a secondary condition, the likelihood of all four specific lameness diagnoses—foot rot, joint infections, lameness without visible swelling, and injuries—remained unchanged. This finding suggests that the behavioral alterations and potential immunosuppression associated with BRD do not necessarily correlate with an elevated risk of lameness [110].

#### 3.3.4. Some Prospective Studies in Behavior Changes in BRD Diagnosis and Prognosis

The investigation into behavioral changes due to BRD warrants careful analysis to ascertain the impact of both internal factors—such as genetic predisposition, age, immune status, and overall health—and external factors—such as housing conditions, environmental stresses, social interactions, and management practices—alongside the disease’s own influence on the behavior of calves exhibiting clinical or subclinical symptoms. For precise assessment of these behavioral modifications, dependable diagnostic procedures for BRD are essential. Research indicates that while there may be variability in pulmonary inflammation, systemic inflammation appears comparably consistent in both non-diseased and subclinically affected calves [111]. Studies have documented a reduction in exploratory behavior, rumination, hay consumption, and self-grooming in calves experiencing clinical disease or when subjected to a bacterial endotoxin lipopolysaccharide (LPS) challenge, as opposed to their healthy counterparts [88,112]. Exploring the potential link between inflammatory cytokine levels, behavioral patterns, and BRD prevalence through the integrated application of lung ultrasound techniques and clinical respiratory evaluations may shed light on the origins of clinical symptoms in calves impacted by BRD.

#### 3.3.5. Behavior Changes and BRD Treatment by Antibiotics and NSAIDs

The impact of BRD treatments on animal behavior has undergone investigation using an inducible BRD model. Researchers evaluated the effectiveness of meloxicam in treating BRD, concluding that the exclusive use of NSAIDs is not advisable for BRD management either before transport or upon arrival at the destination [50]. The study found no behavioral or clinical differences between cattle treated with NSAIDs and those that were not. However, variations in BRD severity, as determined by clinical scores, may have obscured the detection of any beneficial or detrimental effects of the treatment [50]. Following Draxxin administration, a 7-day moratorium was observed to maintain therapeutic antibiotic levels within the animals’ tissues to combat BRD [113]. Cattle were visually inspected at least daily for BRD symptoms, such as depression, lethargy, anorexia, respiratory difficulty, and eye or nasal discharge. Affected individuals were isolated for detailed examination [114]. The benefits of combining NSAIDs with antibiotics to treat BRD have been inconclusive [115]. While some studies report positive outcomes like reduced lung consolidation and better overall performance, others do not show significant improvements in disease progression or performance.

Further research assessed the relationship between feeding behavior, activity levels, and recovery in dairy calves treated for BRD [116]. Over a 10-day observation period post-treatment, calves that recovered demonstrated increased starter feed intake and heightened activity compared to those that relapsed, which displayed reduced feeding and extended periods of lying down. These findings indicate that precision technology devices, such as automatic feeders and pedometers, could be pivotal in gauging the recovery status of calves post-BRD treatment. Continuous monitoring of feeding and activity patterns may enable the early identification of calves at risk of relapse before clinical signs reappear. This study underscores the value of precision technology in evaluating the health status of calves with BRD, potentially reducing disease duration and enhancing calf welfare and health overall.

### 3.4. Necropsy as a BRD Gold Standard Diagnostic Test

A postmortem examination, coupled with diagnostic testing for agents of BRD, is recognized as the definitive method for accurately diagnosing BRD in calves. BRD is a significant contributor to mortality among cattle during live export, with two-thirds of necropsy samples from 20 long-distance shipments (*n* = 130/195) confirming infectious lung disease [117]. Necropsy serves as a tool to evaluate the accuracy of veterinarians in detecting lesions and pinpointing specific infections. It has also been instrumental in studies assessing the efficacy of thoracic ultrasonography (TUS) for diagnosing chronic [84], subclinical BRD [111], and infections induced by *M. haemolytica* [118]. Despite its utility, necropsy is not commonly employed for BRD diagnosis unless an outbreak reaches epidemic levels within a herd [22,119]. The method of examining the right lung is preferred over the left due to its higher reliability in detecting primary lung disease [120,121]. Nonetheless, challenges such as false negatives in early-stage BRD or viral pneumonia—where lung consolidations are absent—and false positives from prior, resolved BRD episodes do occur. Additionally, examining only the right lung could overlook up to 16% or even 30% of lesions [83,122]. Efforts to innovate diagnostic methods include developing image classification models through machine learning. These models are trained on images of necropsied right lateral lungs annotated with gross diagnoses. Although they have demonstrated moderate sensitivity in detecting BRD, there is a need for further refinement to enhance their diagnostic accuracy [123].

Enhancing BRD detection also involves analyzing necropsy data to determine the cause of death and the proportion of calves succumbing to undiagnosed respiratory diseases. Extensive research has identified key respiratory pathogens via gross necropsy and histopathologic examination [13,124,125]. However, given the variable time between disease onset and mortality—ranging from days to weeks—relying solely on autopsy findings and pathogen isolation may not suffice for early detection and prevention of BRD. Postmortem testing offers advantages, with many studies employing virus isolation, fluorescent antibodies, immunohistochemistry, and virus culture from samples collected at necropsy or from airway swabs [7,125]. These studies have provided detailed insights into the pathogens affecting livestock. Yet, the diagnostic tests currently available struggle to distinguish between naturally occurring field strains and those derived from vaccines.

Comparisons between pre-mortem diagnoses and post-mortem examinations in dairy calves that died before reaching 90 days of age showed modest agreement, with Cohen’s kappa indices of 0.22 and 0.13, indicating slight to fair concordance [126]. An analysis encompassing seven studies disclosed that pen riders in feedlots identified only 3–56% of cattle with respiratory illnesses at the time of slaughter [72]. In a study of adult cattle mortality in western France, BRD was identified as the second leading cause of death following digestive disorders. The primary lung lesions associated with BRD often included infectious primary pulmonary lesions (IPP) and thromboembolic pneumonia (TEP), with fibrinous hemorrhagic and/or necrotic bronchopneumonia being the most prevalent IPP lesion, and *M. haemolytica* emerging as the predominant pathogen [127]. While necropsy and identification of BRD pathogens remain the definitive methods for diagnosing BRD, clinical scoring systems offer a valuable alternative for categorizing affected cattle, especially for individuals lacking specialized expertise [24].

The occurrence of BRD in feedlot calves aligns with an average prevalence rate of 17.0%, although estimates range from 4.6% to 43.8%. This rate surpasses those documented in countries such as the Netherlands, Italy, and France, where prevalence is reported below 7%. Notwithstanding these figures, the actual incidence of BRD may be higher than reported since one-third of calves exhibited extensive pneumonia at necropsy without a prior diagnosis [128,129].

In terms of BRD pathogens, the detection rate of bovine coronavirus (BCV) in lung tissue during necropsy was typically low, suggesting BCV may act more as a predisposing factor for secondary lung infections rather than as a primary respiratory pathogen [117,130]. Meanwhile, BVDV antigens were frequently found in the respiratory tracts of animals with BRD [131]. BVDV genomes were also detected in the transtracheal washes of both slaughtered and necropsied animals, some of which showed no clinical signs [132]. It is common for animals with severe pneumonia to succumb to the disease, particularly those with significant fibrinous pleurisy found at necropsy [133]. The role of viral infections in the development of histophilosis remains uncertain when viruses are absent at the time of death, even though *H*. *somni* has been implicated in causing pneumonia independently [133,134,135].

When culturing lung tissue from necropsies, similar challenges may arise due to previous antimicrobial treatments, which can lead to increased levels of antimicrobial agents and the presence of resistant bacterial strains in the tissue [136]. Notably, antibiotic susceptibility testing of isolates from lung tissue can yield valuable insights into the infection site. However, this is influenced by factors such as the timing of sample collection, prior exposure to antimicrobials, and concurrent bacterial infections. Moreover, the effectiveness of treatment protocols for cattle may be affected by the inherent prevalence of antimicrobial resistance within the bacterial microbiome or resistance gene expression following antimicrobial therapy [137].

A significant number of adult cattle that underwent necropsy were found to have succumbed to fatal pneumonia [138,139,140]. This complicates the establishment of an accurate standard reference diagnostic test that could prevent misclassification of the disease [21]. In response to this challenge, three alternative strategies may be considered: (i) choosing a surrogate marker for the condition in question, despite potential bias due to its limited accuracy (e.g., TUS for BRD); (ii) employing a composite reference test, which carries its own risk of bias [141]; and (iii) acknowledging the constraints of any field tests when determining the true health status of the animals [142].

## 4. Imaging Techniques for BRD Diagnosis and Prognosis

### 4.1. Thoracic Radiography

During the 1980s, researchers observed that chest radiographs of asymptomatic calves could reveal pulmonary lesions [143]. Subsequent studies detailed the pathogenesis of infectious bronchopneumonia in calves and cows, often presenting as alveolar opacification with or without visible air bronchograms [35,144]. Additionally, cavitary lesions associated with abscesses and emphysematous bullae were identified [35]. Other radiographic patterns, such as thickened bronchial walls, interstitial markings, and the summation effect resulting from the overlay of three-dimensional structures onto two-dimensional images, were also documented [86]. Despite these advances, the application of thoracic radiology in veterinary practice faces challenges, including the limited availability of specialized veterinarians, radiation exposure risks, and the need for comparative analyses with other imaging modalities. A study involving 50 hospital-admitted calves weighing under 100 kg showed that the diagnostic accuracy of thoracic radiographs and ultrasonography was similar to that of computed tomography (CT). Although CT remains a benchmark in human pneumonia research for detecting mild to moderate lung changes, its prohibitive cost and requirement for anesthesia render it impractical for widespread veterinary use [35]. Diagnostic imaging techniques such as chest ultrasound and X-rays are valuable for the antemortem diagnosis of BRD, but their utility is constrained by the cost of equipment and the expertise required for accurate interpretation [83]. To address these limitations, researchers have explored computer-aided lung auscultation (CALA) using the Whisper Veterinary Stethoscope (Merck Animal Health, Madison, New Jersey, USA) [145]. This system facilitates the diagnosis and treatment of BRD by wirelessly transmitting thoracic sound recordings to a computer for analysis. The resultant CALA score, ranging from 1 to 5, indicates the severity of lung pathology and supports timely clinical decision-making. By automating auscultation with a proprietary machine learning algorithm, CALA offers objective assessments of lung health. Its ability to rapidly and quantitatively evaluate pulmonary conditions provides a valuable tool for early detection and precise management of BRD, potentially reducing reinfection rates and mortality in affected cattle populations [145].

### 4.2. Thoracic Ultrasonography of the Chest for BRD Diagnosis, Prognosis, and Treatment Follow-Up

#### 4.2.1. TUS in BRD Diagnosis and Prognosis: Sensitivity, Specificity, and Probes Used

Recent assessments of TUS have confirmed its utility for clinical purposes. This technology provides a non-invasive and expedient means to detect pulmonary abnormalities on-site. Research indicates that TUS is a dependable method for assessing lung consolidation, even when performed by individuals with minimal experience [32,86,146,147]. Nevertheless, the technique is limited by its inability to image the lung parenchyma above the heart in large beef calves due to their substantial size and muscle mass. Distinguishing between active, treatable lung lesions and those resulting from prior, treatment-resistant conditions remains a significant challenge [30,148,149,150]. Diagnostic imaging plays a crucial role in differentiating normal from pathological findings [86]. Berman et al. (2019) reported that TUS achieved an 89% sensitivity and 95% specificity for detecting consolidations deeper than 3 cm, excluding the area above the heart, in a study involving 209 calves and 30 pre-weaned dairy calves. This study used WRSC and serum Hp levels as benchmarks. Despite the observational nature of pulmonary lesion imaging studies, no data suggests these procedures enhance overall health or productivity [151]. Zeineldin et al. (2016) examined the use of TUS between the 7th and 11th intercostal spaces (ICS) in 6–8 month-old veal calves, discovering that it effectively identified diverse echogenic lung areas. The technique demonstrated a sensitivity of 70.8% and a specificity of 87.5% [152]. In addition, Ollivett et al. (2015) evaluated TUS in 25 dairy calves aged 1–12 weeks, using necropsy as the benchmark. Their findings indicated that TUS had a high sensitivity (94; 69–100) and specificity (100; 64–100) for detecting lung lesions related to BRD in asymptomatic calves [111]. Similarly, Rabeling et al. (1998) investigated the use of TUS in calves up to 5 months old, presenting with arthritis or chronic lesions at a veterinary clinic, resulting in a sensitivity of 85% and a specificity of 98% [84]. Cumulatively, these studies suggest that TUS is a reliable method for identifying pulmonary consolidation in calves, irrespective of their clinical status [153].

The 3.5 MHz sector ultrasound transducer is widely utilized in ultrasound imaging to identify various conditions, including lesions, abscesses, and thoracic fluid. A study explored the caudal lungs and the parenchyma located cranially to the heart on the right side using this transducer [86]. Presently, many veterinarians prefer the use of 7.5 MHz linear rectal probes due to their slender design that facilitates insertion between ribs. Research suggests that this method can provide a precise diagnosis of pneumonia and prove particularly valuable for examining calves, whose forelimb musculature often obstructs access to the cranial thorax. To discern lung consolidation, the most common approach involves identifying a hypoechoic region with echogenicity similar to that of liver parenchyma. A weekly assessment over the first 56 days of life in 123 female Holstein calves compared the frequency distribution of lung consolidation detected by TUS with findings from postmortem examinations [149]. Findings revealed that out of 24 dairy calves aged under 12 weeks, 6 calves exhibited lung consolidation, predominantly in the middle and cranial lobes. In buffalo calves, TUS identified pulmonary comets, which are indicative of pathology near the pleural line edges in the images [30]. Physical examinations in tandem with TUS are advocated for evaluating pre-weaned dairy calves exhibiting symptoms of respiratory diseases [154].

Inadequate penetration of lung consolidation into peripheral regions may compromise the accuracy of lung ultrasound results. Moreover, the scoring system employed to gauge the intensity of feeding patterns lacks precision [27]. Under certain conditions, calves with non-aerated lungs from causes other than BRD may be incorrectly diagnosed as positive [27]. A study utilizing Bayesian estimation to assess the effectiveness of clinical respiratory scoring (CRS) and lung ultrasound concluded that with a cutoff point of one, the sensitivity of CRS was 62.4% (95% CI: 47.9–75.8), and its specificity was 74.1% (95% CI: 64.9–82.8) [43]. At a cutoff of one centimeter or more, lung ultrasound demonstrated a specificity of 93.9% (95% CI: 88.0–97.6%) and a sensitivity of 79.4% (95% CI: 66.4–90.9%) [90].

TUS and thoracic radiography (TR) were found to be similarly effective in detecting thoracic lesions. Despite this, prior research indicates that TUS has a lower sensitivity than TR for diagnosing respiratory conditions in neonates [155]. Nevertheless, TUS has shown promise in accurately identifying thoracic lesions in dairy calves. For more robust validation of these findings, further research with a larger sample size (exceeding 400 calves) and adequate statistical power is recommended [35].

#### 4.2.2. TUS as a Tool for Treatment Follow-Up and the Disease’s Recovery

TUS, a non-invasive imaging technique, is utilized to identify lung lesions indicative of pneumonia and pleurisy. A positive TUS finding is defined by the presence of consolidation less than three centimeters caudal to the heart without assessing the cranial side [51,156].

During the treatment of BRD in calves, the progression of ultrasound lung scores is useful for monitoring the recovery of lung tissue [51]. Despite this, research on the accuracy of TUS for diagnosing active pneumonia, rather than merely detecting lung lesions, remains insufficient. It is crucial to consider that calves with previous pulmonary infections may retain chronic inactive lesions, which highlights the importance of discerning between active infection and residual damage when utilizing TUS to determine the necessity for treatment.

TUS offers a somewhat imprecise yet objective method for assessing various BRD treatments. The use of tildipirosin as a metaphylactic agent was shown to be ineffective in reducing the prevalence of ultrasound-detected lung lesions at weaning, indicating no reduction in the incidence of BRD [157]. Despite this, calves treated with tildipirosin exhibited improved lung health upon subsequent TUS examination and after pathogen challenge. Comparisons between animals administered tildipirosin or tulathromycin and those untreated (negative controls) revealed significantly lower lung lesion scores in the tildipirosin group [158]. Additionally, administering tildipirosin five days before exposure to *H. somni* resulted in milder lung lesions, absent necrosis, and confinement to areas of acute bronchopneumonia amidst otherwise healthy lung tissue [159].

#### 4.2.3. The Pros and Cons of TUS as a Promising Tool for BRD Diagnosis and Prognosis Research

Ultrasonography (TUS) values are frequently detectable at low levels, even in the absence of clinical signs and symptoms. This imaging modality has become indispensable in both research and clinical settings, including referral hospitals, and is increasingly utilized in primary care. TUS proves capable of identifying early disease stages and asymptomatic cases, facilitating the initiation of early treatment. Moreover, it can be employed to predict recovery timelines. In the UK, the integration of TUS on farms has substantially enhanced disease surveillance, resulting in a heightened detection rate and a more precise diagnosis of BRD. Given the constraints associated with postmortem examinations, there is a pressing need to establish a more robust correlation between TUS findings and pulmonary pathology. While pulmonary pathology remains the definitive diagnostic approach, its requirement for euthanasia renders it less favorable. In contrast, TUS presents a non-invasive alternative that has demonstrated results closely mirroring gross pulmonary pathology, particularly when examining an extensive portion of the lung field (Table 1). This correlation implies that TUS could effectively substitute more invasive diagnostic methods.

TUS is recognized as a highly reliable reference method for respiratory disease studies, offering advantages over alternative assessments like the Wisconsin Respiratory Score and cytologic and bacteriologic analyses of bronchoalveolar lavage specimens [90,160,161]. Its effectiveness in differentiating between upper and lower respiratory tract diseases, as well as distinguishing infectious from non-infectious origins, is well-documented [148,149]. TUS is linking lung consolidation to delayed growth, increased mortality, lower reproductive performance, and a shorter life expectancy. For instance, studies on preweaning dairy calves have employed TUS to identify lung consolidation, noting a significant correlation with reduced ADG [59]. However, the application of TUS to assess the effects of BRD on housed beef cattle, specifically concerning clinical respiratory symptoms and lung consolidation, has not been extensively investigated.

The use of TUS extends beyond clinical diagnosis; it serves as a non-invasive tool to monitor disease progression during both field and controlled study infections. The use of TUS extends beyond clinical diagnosis; it serves as a non-invasive tool to monitor disease progression during both field and controlled study infections. Particularly valuable for identifying illnesses that may not be apparent during clinical examinations, TUS offers insights into the pulmonary health of calves. Although the exact duration of disease resolution and its underlying mechanisms are not yet fully understood, evidence suggests that the decline phase outlasts the active infection period. Investigating the resolution phase of BRD could profoundly impact the understanding of pathogenesis and long-term animal performance.

Recent findings highlight the association between higher TUS scores and decreased ADG, particularly in chronic cases, which are more severe than indicated by clinical respiratory assessments alone. A comprehensive study involving 317 calves across seven farms over six weeks generated a dataset of 966 TUS and CRS values. This dataset was used to construct two multivariable linear mixed-effects regression models to analyze the relationship between TUS scores, CRS values, and weight changes. The Calf Respiratory Score Chart from the University of Wisconsin facilitated the reporting of CRS values, while a specialized weighing tape for dairy cattle breeds was utilized for live weight measurements [162].

**Table 1:** Studies on the Use of TUS for BRD Diagnosis and Prognosis: Objective of Studies and Key Findings.

Objective	Study Design	Sample Size	Date of the Published Study	Location	Key Findings	Reference
Assessing the diagnostic and prognostic utility of lung ultrasonography in BRD	longitudinal design	600	2023	Austria	High sensitivity (86%) and specificity (78%)	[163]
Assessing TUS’s diagnostic and prognostic value in initial BRD cases	Prospective longitudinal study	174	2012	Canada	TUS proved valuable in targeted populations of animals with prolonged respiratory disease.	[29]
Improving BRD detection in experimental infection using TUS	Experimental Study	62	2021	USA	TUS is a rapid predictor of lung lesions in pre-weaned dairy calves. TUS can identify calves with clinical and subclinical pneumonia with high sensitivity. TUS detects abnormal lung pathology missed by clinical scoring alone. TUS score correlates strongly with gross lung pathology on necropsy	[164]
Assessing the impact of lung consolidation detected via TUS on health and growth outcomes in BRD	Longitudinal Study	221	2023	Iran	The sensitivity ranges from 86% to 94% and the specificity ranges from 98% to 100%. Additionally, one or more consolidation episodes resulted in a significantly lower ADG.	[165]
Assessing subclinical lung lesions in Holstein calves using TUS and bronchoalveolar lavage fluid analysis.	Prospective study	25	2015	Canada	TUS had a 94% sensitivity and 100% specificity in detecting lung lesions related to subclinical BRD in healthy calves.	[111]
Evaluating the accuracy and inter-rater reliability of lung auscultation among bovine practitioners relative to TUS findings	Diagnostic test study	49	2019	Netherlands	TUS is the most accurate for practical use in the field	[166]
Assessing the association of clinical respiratory signs and lung lesions detected via TUS with growth performance in pre-weaned dairy calves	Retrospective cohort study	53	2021	Ireland	TUS found lung consolidation in healthy calves, and severe lung lesions affected their pre-weaning growth.	[67]
Assessing the efficacy of TUS for diagnosing BRD in preweaned dairy calves	Longitudinal study	60	2023	USA	The sensitivity and specificity of TUS vary depending on the scoring method used.	[167]
Evaluating the efficacy of lung ultrasonography and clinical assessments in monitoring BRD in fattening bulls during restocking and post-treatment with tulathromycin and ketoprofen	case time-series analysis	60	2022	Italy	TUS presents a higher sensitivity (80–94%) and specificity (94–100%) than clinical scoring to detect lung lesions. It is a non-invasive and cost-effective tool for BRD early diagnosis and for monitoring treatment efficacy.	[51]

### 4.3. Focus Lung Ultrasonography as a Tool for Evaluating Lung Lesions in BRD

Focused Lung Ultrasonography (FLUS) is a medical imaging technique utilized to evaluate lung conditions. Clinical data reveal that FLUS has a sensitivity of 81.6%, specificity of 100%, positive predictive value of 100%, negative predictive value of 96.6%, and an accuracy rate of 97%. These metrics demonstrate substantial concordance with TUS, as evidenced by a weighted kappa value of 0.78 [168]. However, FLUS has certain limitations in detecting lung lesions associated with BRD. It serves best as an adjunctive diagnostic tool for identifying consolidations, especially beneficial when conducting mass screenings in post-weaning dairy calves. FLUS is tailored to assess regions commonly affected by BRD, including the caudal aspect of the cranial lobe of the left lung, the right lung’s middle lobe, and the caudal aspect of the cranial lobe of the right lung [168].

## 5. Molecular and Sample-Based Methods for BRD Diagnosis and Prognosis

### 5.1. Molecular Diagnostics

Given the diverse array of pathogens associated with BRD, molecular diagnostics targeting unique genetic regions of these pathogens have become a promising approach for diagnosis. Quantitative Polymerase Chain Reaction (qPCR) and Reverse Transcription qPCR (RT-qPCR) are commonly used as benchmark diagnostics in the absence of an established gold standard [169,170]. For the detection of both viral and bacterial pathogens linked to BRD, diagnosticians frequently employ qPCR assays [171]. Additionally, other molecular techniques, including 16S rRNA gene sequencing and next-generation sequencing, alongside qPCR, have been utilized in analyzing the nasal microbiome of BRD cases. These methods aim to discern variations in microbial composition and abundance [46,172]. These assays necessitate rigorous adherence to laboratory protocols and often require costly equipment to guarantee reliable and actionable results [173]. Consequently, there is a demand for alternative diagnostic methods that simplify the training process for users and expand the potential locations for conducting these assays.

Isothermal amplification techniques represent a category of methods that reduce the need for highly skilled personnel and laboratory infrastructure. These techniques are conducted at a constant temperature and typically involve a single reaction container, simplifying both the complexity of the reaction and the necessary training and equipment for its execution [174]. Mohan et al., demonstrated the effectiveness of Loop-mediated Isothermal Amplification (LAMP) for identifying BRD bacterial pathogens in bovine nasal swabs using conventional laboratory apparatuses [175]. Subsequently, Pasucal-Garrigos et al. expanded upon this approach to create a colorimetric assay in liquid solution, which induces a visible color change in unprocessed nasal samples under field conditions with a basic heater [176]. Furthermore, LAMP has been adapted to paper-based devices, enhancing manufacturing efficiency and ease of use [177,178]. Despite these developments, current literature indicates that the application of LAMP on paper-based devices for detecting BRD pathogens remains unexplored.

### 5.2. Diagnosing BRD through White and Red Blood Cells as Well as Blood Plasma

BRD is linked to systemic changes observable in various body fluids, with blood being the primary focus of observation. Studies on the diagnostic efficacy of white blood cell (WBC) alterations due to respiratory infections are extensive, yet they reveal limited utility [34]. For instance, a recent study examining blood cell accuracy in dairy calves reported a sensitivity of 46% and a specificity of 82% [34].

The evaluation of both white and red blood cells in calves affected by naturally occurring respiratory diseases demonstrated comparable results for the area under the receiver operating characteristic curve (AUC). The AUC for basophil count, identified as the most reliable WBC marker, was only 0.599, signifying its low diagnostic accuracy [179], suggesting that WBC results are of limited utility in diagnosing BRD. Moreover, correlations between total neutrophils (with PCLD), segmented neutrophils (with LLD), leukocytes (with LLD and PCLD), plasma levels (with LLD), and lung consolidation percentage were not significant, which could be attributed to the small sample size used in the study. It is recommended that future research includes a larger cohort to better understand the relationship between leukocyte differentials and lung consolidation [180]. Moreover, the lymphocyte count, total neutrophil count, and neutrophil-to-lymphocyte ratio demonstrate alterations before and after bacterial provocation, coupled with augmentations in band neutrophils and fibrinogen over time. In combination with their relationship to lung consolidation, such shifts may be used in predicting BRD progression and viral and bacterial infections [180].

The spectral profiles of near-infrared spectroscopy (NIR) from dairy calves’ plasma infected with *M. haemolytica* were characterized and validated against standard clinical and hematological reference values. Baseline blood samples were collected four days prior to the controlled intrabronchial challenge, with subsequent samples obtained 23 days post-infection. PCA-LDA models facilitated the identification and prediction of deviations from normal baseline to infection states by comparing NIR blood plasma spectral profiles.

The study revealed that NIR spectral profiles reflect biochemical and physiological changes in dairy calf blood plasma during *M. haemolytica* infections. This finding suggests that NIR spectroscopy could be employed for the rapid detection of BRD caused by *M. haemolytica* within a diagnostic setting [181]. While hematological parameters alone are insufficient for a definitive diagnosis of BRD, variations in these values can aid in the diagnosis, monitoring of disease progression, and prognostication. [182,183]. Furthermore, the presence of neutrophils in the lung secretions of Holstein calves at concentrations below 4% has been linked to pulmonary consolidation, providing an additional diagnostic parameter for assessing the health status of calves affected by respiratory disease [111].

### 5.3. Transcriptomic and Metabolic Profiling in BRD Diagnosis

The expression of various immune-related genes was evaluated at the sites of BRD pathogen infection, including bronchial lymph nodes [184,185], lung tissue [186,187,188], and lymph fluid [189]. The results revealed significant transcriptional changes in the whole blood of infected animals compared to healthy and market-ready counterparts [190], with an upregulation in genes associated with the inflammatory response and a downregulation in genes involved in cell metabolism, growth, and maintenance. qPCR could be performed on a larger sample of animals to further validate these studies [191]. In artificially reared dairy calves infected with BRSV, systemic changes in gene expression occurred in the absence of clinical or pulmonary symptoms. These alterations could be more accurately quantified by examining easily obtainable tissues like whole blood [192]. Notably, in deceased cattle, heightened EGs linked to innate immunity, even without detectable viral genes, points to the significance of antiviral defenses and type I interferon signaling in BRD susceptibility, laying the groundwork for further exploration into its pathogenesis and the role of antiviral mechanisms in mortality [193]. Post-weaning whole blood transcriptomes were compared from cattle that developed BRD within the first 28 days after arrival to those that did not. The transcriptomes included specific genes such as MCF2L, MARCO, CFB, LOC100335828 (CD200R1), ALOX15, and SLC18A2. However, biological variability among individual cattle and populations presents a significant challenge due to factors that can affect transcriptome data. Additionally, the lack of comprehensive pre-arrival treatment and vaccination records for these populations compounded this challenge. This variability was further complicated by the inclusion of commercially raised cattle with unknown genetic backgrounds [193,194]. A major constraint of this study was the insufficient power to identify genes with differential expression capable of predicting BRD occurrence within 28 days post-arrival. Future research should integrate factors that influence transcriptome variation across beef production systems into experimental design to facilitate the identification of correlations between gene expression, disease manifestation, and sources of variation. Despite these limitations, ROC curve analysis provided preliminary evidence supporting the potential validation of predictive biomarkers for early detection of BRD. For more conclusive results, these methods should be applied to larger bovine populations to enhance the statistical power necessary for identifying genes associated with BRD progression [195]. Another investigation employed NanoString nCounter gene expression profiling to assess mRNA expression patterns upon arrival in beef cattle with BRD. This profiling revealed differentially expressed genes involved in immune responses, proinflammatory processes, granulocytic activities, and type I interferon signaling, suggesting potential markers for forecasting the development and severity of BRD [196]. Significant variations in microRNA (miRNA) expression were observed in the lymphatic system tissues of calves challenged with either *M. bovis* or a combination of *M. bovis* and BVDV, with BVDV resulting in a reduction of miRNA counts. In contrast, no significant differences were found in white blood cells, and only limited differences were detected in serum [197]. Furthermore, specific genes and pathways implicated in the immune response to BHV-1 infection were identified, offering potential biomarkers for BRD diagnosis and prognosis. These findings were also compared with those from BRSV infection, revealing both shared and unique immune responses that provide insights into the host’s reaction to different BRD pathogens [198]. It is crucial to acknowledge that despite observing differential gene expression across ages, no significant differences were detected in peripheral leukocyte gene expression between diseased and healthy pre-weaned Holstein heifer calves diagnosed with respiratory disease [199].

Gene expression signatures have been recognized as potential biomarkers for BRD diagnosis and prognosis. These biomarkers facilitate the early detection and monitoring of disease progression. Moreover, they provide insights into the impact of marketing decisions, such as commercial auctioning and direct transportation, on the development of BRD. This knowledge supports risk assessment and the implementation of targeted preventive measures [200].

Furthermore, blood metabolome analysis has proven highly accurate in predicting BRD when applying a visual diagnostic method. Specifically, 85% of animals in the validation dataset were correctly classified as BRD-affected or healthy. This accuracy rate is marginally lower than the 95% reported in a prior study, which had a smaller sample size consisting of 50 BRD animals and ten controls [201,202]. The authors attributed the higher metabolomics accuracy in that study to the use of advanced statistical techniques and experimental design, including the use of only 10% of the validation data as predictors, rather than relying on component or peak analysis [201]. Metabolomics analysis of the plasma metabolome in beef steers over a 35-day receiving period identified changes in cysteine and methionine metabolism. Additionally, there were observable variations in the plasma concentrations of sarcosine, methionine, dimethyl sulfone, and L-histidine between BRD-affected and healthy groups. These findings suggest potential biomarkers for BRD and targeted interventions to mitigate its adverse effects on the health and performance of beef cattle [203]. In another study, specific metabolites such as 2-hydroxybutyrate, acetone, and 3-hydroxyisobutyrate, identified through nuclear magnetic resonance (NMR), were found to be significantly altered in plasma samples from infected calves compared to controls. These metabolites present promising targets for the development of diagnostic methods for the early detection of BRD [204].

### 5.4. Acute Phase Proteins in BRD Diagnosis and Prognosis

The production of APPs such as Hp in the liver is a direct response to inflammatory cytokines like TNF-α and IL-1β, thus differentiating between acute and chronic inflammation [1,205]. Serum Hp levels can detect early-stage metritis and serve as a diagnostic marker for inflammation in BRD [28,34,206,207]. Research by Grell et al. showed increased Hp, IFN-γ, and IL-6 in animals post-BRSV infection, and Heegard found higher Hp levels in infected animals versus controls [208,209]. Wernicki et al., suggested that Hp could be a reliable marker for identifying respiratory diseases caused by transport stress in veal calves [210]; however, its diagnostic reliability for respiratory diseases in cattle is questioned due to variable accuracy and a limited number of studies. A notable interaction was observed between treatment days and increased Hp levels, with day 9 showing the strongest association with BRD, suggesting that many cattle had not been previously exposed to respiratory pathogens [211].

To address these limitations, alternative tests, including blood gas analysis, respiratory biomarkers, and analysis of respiratory secretions have been developed. Serum iron concentration has emerged as a potential substitute for Hp and serum amyloid A (SAA) in diagnosing respiratory diseases in cattle [212]. It is essential to identify new biomarkers to accurately assess an individual calf’s risk of developing BRD and improve the management of this condition [31,213,214,215]. There were no detectable differences between the treated and untreated animals for multiple measured analytes. However, the origin of the barn appears to have affected TNF-α levels. Additionally, the differences in all analytes changed over time, correlating with treatment on certain days.

Comparative studies on other APPs like serum amyloid A, fibrinogen, and C-reactive protein (CRP) for diagnosing bovine respiratory infections have produced variable results in their accuracy, sensitivity, and specificity [216]. A meta-analysis was not feasible due to research design disparities, site variations, and differing case definitions, leaving their effectiveness in detecting BRD uncertain [34].

Procalcitonin, an additional APP, has proven valuable for diagnosing and prognosticating BRD; however, its specificity extends beyond BRD-related bacterial infections to systemic inflammatory responses like sepsis and mastitis [217,218].

Other potential indicators include total protein concentration in serum as a BRD predictor in newborn calves, although data on the relationship between neonatal BRD and protein metabolism indicators is scarce [219,220]. Previous studies have demonstrated that Beta defensins can be induced by *M. haemolytica* infection, especially in cases of subacute or chronic infections [221]. Moreover, serum iron concentration may offer a simple and cost-effective alternative to APPs for evaluating inflammatory diseases [222]. Some of the available biomarkers for BRD diagnosis and prognosis (Table 2). 

## 6. Automated and Predictive Methods for BRD Diagnosis and Prognosis

### 6.1. Automated Behavior Detection

Recent studies have implemented bi-weekly calf health assessments to improve the evaluation methods used in behavioral research [87,89,93,99]. These assessments incorporated the Wisconsin CRS chart [229] and lung ultrasonography [86], enabling the automatic identification of changes in feeding behavior during the three days preceding and on the day of BRD detection in calves. This allowed for distinguishing between subclinical BRD (characterized by a negative CRS score and lung consolidations ≥ 1 cm^2^), clinical BRD (positive CRS score with or without lung consolidations), and healthy calves (negative CRS score and lung consolidations < 1 cm^2^).

The collected data from non-nutritive visits revealed that calves with poor weaning outcomes displayed a decreased drinking rate and shorter suckling times [104,230]. While automated systems can detect these behavioral shifts earlier than human observers, the risk of false positives remains [104]. Although these metrics can track the daily feeding patterns of dairy calves using milk vending machines, their reliability in predicting illness is not yet proven [104]. A significant limitation of automated detection is its comparison to a clinical definition that, while deemed best at the time, is now one among many, each with its flaws. Since clinical diagnosis is typically the benchmark in field studies, any mismatch between testing criteria and case definitions can compromise the accuracy of results.

The remote early disease identification (REDI) system, coupled with a Bayesian latent class model [231,232], utilized real-time animal position data to monitor feedlot calves. With no gold standard for identifying clinical cases, the REDI system proved more accurate than manual checks by barn staff. Research indicated that treatment was more successful and required fewer antibiotic treatments per animal when employing this system for respiratory infection detection [232].

The effectiveness of visual monitoring for BRD diagnosis is influenced by factors such as social dynamics, environmental conditions, work schedules, and the observer’s skill level. The proficiency of the pen rider is particularly critical; a lack of expertise may result in erroneous BRD diagnoses within large herds [103]. However, advancements in technology have facilitated continuous individual animal monitoring, with sensor use becoming increasingly prevalent on dairy farms for heat detection and rumen activity monitoring—yet not universally adopted within the bovine population [233]. Predictive modeling based on precision farming data has proven highly effective in early BRD diagnosis in pre-weaned calves.

Studies has shown that the behavior of cattle can reveal valuable information about BRD. For instance, Growsafe in Airdrie, Alberta, Canada, observed that during natural BRD outbreaks, there were noticeable differences in the individual feeding patterns of infected cattle compared to their healthy counterparts. Studies by Buhman et al. and Sowell et al. noted that cattle with BRD exhibited shorter durations at the feedlot, ranging from 1–4 days and 11–27 days, respectively [234]. Although this research underscored daily behavioral changes, it likely missed more nuanced variations occurring over shorter timeframes. Consequently, there is an imperative to develop diagnostic tools capable of detecting these finer daily fluctuations. Advanced models, like Random Forest (RF), may be effective in differentiating the subtle feeding behaviors of clinical versus subclinical BRD cases; thus, further investigation is warranted [235]. Nonetheless, relying solely on the rate of drinking as a diagnostic measure for disease detection is inadequate. Yet, when integrated with additional feeding behaviors, it becomes a powerful method for identifying sick calves.

Studies assessing changes in drinking behavior around BRD diagnosis revealed that calves consuming an average of 9.4 L of milk per day experienced a marked decline in drinking speed on the day they were diagnosed with BRD and showed a drop in unrewarded visits to the feeder ten days preceding the diagnosis [104]. A similar decrease in unrewarded visits occurred two days before BRD diagnosis in calves consuming 6 L or 8 L of milk/day [236]. Delayed detection of BRD can impair treatment effectiveness and outcomes [237]. Modern technology allows for remote, real-time monitoring of physiological parameters, enabling the development of pattern recognition techniques to identify cattle with BRD before physical symptoms become apparent [238]. Moya et al. (2015) found that cows with BRD demonstrated distinctive feeding behaviors compared to healthy ones, illustrating the effectiveness of technology in delivering reliable diagnoses and thereby enabling a more precise evaluation of BRD’s impact on production [105,239].

The link between specific feeding behaviors and a reduced risk of developing BRD up to seven days before clinical symptoms emerge has been established through both discrete survival-time data and feeding behavior metrics. This association indicates the feasibility of developing predictive models for early BRD detection by monitoring feeding behavior changes prior to disease onset. The genomic sequences of *multocida* str. Anand1_cattle (contigs: GCA_000291645.1), *H. somni* [240], and *M. bovis* [241] provide a basis for further exploration into the molecular mechanisms, functions, and networks associated with BRD pathogenesis, which could lead to more accurate predictions of the disease. A thorough understanding of the molecular interactions between host and pathogen is crucial for the precise identification, management, and treatment of BRD. It is also vital to establish behavior models that reflect emergent properties based on the principles of systems biology [186].

Timely intervention can alleviate the adverse effects of various conditions and enhance the effectiveness of treatments [242]. It is essential to evaluate feeding behavior to identify additional economically significant indicators for BRD [85]. The correlation between genetic markers (QTLs) linked to persistent bovine viral diarrhea (BVD) infection and BRD was analyzed. QTLs represent genomic regions linked to specific traits or diseases. The research identified a QTL region 5 proximate to a BRD-related QTL on bovine chromosome 2 (BTA 2) [243]. Furthermore, QTL region 11 and QTL region 18 coincided with BRD-related QTLs on BTA 12 and BTA 26, respectively [244]. A QTL associated with BVD was found to correlate with persistent BVD presence, a virus often involved in BRD outbreaks [245]. The findings also demonstrated that QTLs linked to carcass quality, productivity, reproductive success, and behavioral traits increased in prevalence from QTL region 19 to BTA 20 across various cattle breeds [246].

The utilization of feeding behavior as a predictive marker for BRD prior to its visual diagnosis has been substantiated by the findings of Quimby et al. [96] and Wolfger et al. [85]. These studies found that this method could identify BRD with a sensitivity of 78–82% and a specificity of 78–79% as early as five to six days before the disease could be visually detected. Moreover, clinical assessments revealed that 60–81% of the cattle were indeed suffering from BRD, while 77–85% of the cattle deemed visually healthy were correctly identified as not having the disease [85]. These results robustly support the potential application of this early detection technique. However, to affirm its efficacy, further investigation is necessary to establish the comprehensive sensitivity and specificity of monitoring technologies such as site surveillance systems, pedometers, and accelerometers in the early detection of BRD within naturally occurring cases [66].

#### Role of Accelerometers in Automated BRD Diagnosis

The use of accelerometers and pedometers to detect behavioral changes in cattle with subclinical or mild BRD remains unexplored [238]. These small, non-invasive devices facilitate the remote and objective monitoring of cattle behavior with minimal interference, preserving their natural patterns. Their effectiveness has been demonstrated in analyzing behaviors in beef and dairy cattle, as well as dairy calves. Pedometers complement accelerometers by measuring general activity levels, and this combination has been instrumental in studies investigating the behaviors of cattle experimentally inoculated with moderate BRD symptoms.

Incorporating behavioral indicators, such as time at the feeding bunk, along with periods of lying down and walking into a predictive model [247], enhances the detection capabilities of farm personnel when systematic assessments are not available [87]. Marchesini et al. found that this model’s sensitivity and specificity reached 81% and 95%, respectively, three days prior to the clinical diagnosis of BRD and lameness in cattle [248]. Various studies have utilized pedometers and different types of accelerometers—including tri-axial, ear-tag-based, and electronic—to measure cattle activity levels and identify behaviors indicative of BRD [92,237,249,250,251,252].

### 6.2. Early Disease Detection Systems

For the practical application of these systems to be viable, they must achieve a false-positive rate that is considered acceptable for the early detection of disease. Economic considerations highlight the importance of diagnostic test specificity as a critical factor for feedlot operations. This attribute must be refined to enhance animal welfare, productivity, and economic returns [85]. The emergence of big data and machine learning technologies offers a promising avenue to expedite their deployment, as such tests can monitor a broader range of calf characteristics than human observers can, thus, facilitating the identification of individuals that may need veterinary attention or monitoring by handlers.

The potential benefits of a remote monitoring system capable of detecting BRD up to 0.75 days prior to what is possible through visual inspection are substantial. Such systems can track changes in cattle activity, location, and social interactions, enabling the examination of more animals in less time. This could consequently lead to a reduction in morbidity rates. Moreover, the precision and accuracy of data processing by advanced monitoring systems are enhanced through algorithms and complementary software, which can save both labor and time in commercial fattening operations. Since BRD symptoms are not always apparent through visual assessment, research indicates that statistical process control (SPC) models that include physical activity indicators, such as step count and exercise index, outperform models based solely on visual observations in the early detection of BRD [253]. The implementation of a dual-level BRD scoring system on California dairy farms presents innovative prospects for simultaneous monitoring of BRD in group-housed calves. This scoring system aims to refine the diagnosis of BRD cases and minimize unwarranted antibiotic administration. Its advantages include simplicity in design and lower labor demands, facilitating enhanced early detection with fewer false positives. Therefore, the adoption of such scoring systems is expected to lead to more effective monitoring practices for BRD in the future.

### 6.3. Machine Learning and Modeling Approaches for BRD Diagnosis and Prognosis

The development of predictive models using individual animal data has facilitated the early detection of BRD in calves, effectively distinguishing between healthy and diseased individuals. Previous studies have highlighted differences in feeding and activity patterns between healthy and sick calves [112,237,254]. However, research focusing specifically on BRD detection has been scant. One limitation of these approaches is that the cumulative sum (CUSUM) method does not account for natural behavioral variations over time, such as the decrease in lying duration among aging calves within groups [255]. Comprehensive research is needed to evaluate the accuracy of RF models using expanded datasets. Another constraint of the study pertains to the impact of farm-reported treatments for BRD on the Wisconsin Scoring System. The discrepancy between treatment records and diseases identified by this scoring system suggests a need for additional evaluation. In a study that examined various physiological and behavioral parameters, the most notable outcome was a reduction in activity levels, slower gait speed (*p* < 0.05), and decreased standing duration, as monitored by accelerometers following *M. haemolytica* infection. Pedometers were used to track behavioral shifts in animals clinically diagnosed with BRD compared to control cattle [237].

The use of predictive modeling holds considerable promise in the diagnosis and prognosis of BRD [256,257]. For diagnostic purposes, models can be constructed by combining animal-level, management-level, and herd-level variables to predict the likelihood of BRD in individual animals [256]. These models sift through historical data, detect patterns, and facilitate early detection and precise diagnosis. Prognostically, they take into account factors such as disease severity, treatment response, and chances of relapse to forecast the disease’s outcomes in affected livestock [256]. Incorporating these diverse variables enables veterinarians and researchers to devise more effective treatment strategies and management practices for BRD. However, the potential of predictive models is bounded by certain limitations, including issues with data availability, quality, variable selection, interpretability, and generalizability. The adoption of advanced technologies, such as precision livestock farming (PLF), machine learning, and omics data, offers a pathway to surmount these challenges, enriching the precision and scope of predictive models. Critical to their successful deployment are rigorous model validation, external validation, and the creation of user-friendly decision support systems. By overcoming these hurdles and leveraging new advancements, predictive modeling for BRD can progressively become an indispensable resource for improving animal health, welfare, and management within the livestock sector.

## 7. Utility of Diagnostic Techniques in Prognosticating BRD Outcomes

Timely recognition of BRD poses a significant challenge in veterinary medicine, particularly with subclinical cases that often go undetected, leading to substantial economic losses [83,258,259]. Early treatment of BRD improves the prognosis, whereas delays can result in treatment failure. Enhanced diagnostic specificity could enable more targeted use of antimicrobials, thereby reducing costs associated with BRD management in feedlots [96,97,260]. This is critical as many animals treated for BRD may not actually have the disease, with current clinical diagnosis specificity at 63% [65]. An accurate prediction of BRD during therapeutic intervention is crucial for effective treatment strategies, including drug selection and implementation.

To improve the accuracy of BRD detection and prognosis, novel diagnostic techniques are being adopted. *Pasteurella multocida*, *H. somni*, and *M. haemolytica* are typically implicated pathogens, and antimicrobials are commonly prescribed. However, the rising resistance to antimicrobial agents is compromising BRD treatment outcomes and leading to a poorer BRD prognosis [6].

Rectal temperature has proven to be an unreliable prognostic indicator in calves with suspected BRD [261]. Despite this, an initial rectal temperature over 40 °C is still considered a criterion for improved BRD diagnosis. Visual inspection confirms that rectal temperature alone cannot predict treatment outcomes [261]. TUS offers a non-invasive alternative for the diagnosis and prognosis of pulmonary abnormalities [86]. Additionally, the role of APPs such as CRP in cattle remains underexplored, despite their diagnostic value in other species [262]. Early detection of APPs in serum may occur as soon as 4 h post-exposure in SAA or CRP or later at 24–48 h in Hp or Fb [262]. Previous studies have suggested the diagnostic and prognostic worth of APPs detection in BRD cattle, although the reported results have been variable. Specifically, Hp has proven effective for identifying beef calves with BRD that require treatment and for evaluating the success of therapies administered. Notably, elevated serum concentrations of Hp have been correlated with the presence of *M. haemolytica* and BHV-1 infections in calf BRD model experiments. Consequently, measuring Hp levels is considered useful for diagnosing and monitoring BRD, as well as for assessing the response to treatment [216,263]. However, contrasting studies challenge the reliability of Hp as an indicator for BRD diagnosis in cattle [216,264,265].

While hematological profiling alone cannot definitively diagnose BRD, blood count changes can provide insights into disease detection, monitoring, and prognosis [182,183]. For example, a high neutrophil count ≥4% in bronchoalveolar lavage fluid from Holstein calves correlates with lung consolidation [111]. Automatic cell counters are now routinely used in veterinary practices to aid in the diagnosis and follow-up of systemic diseases.

**Figure 2:**
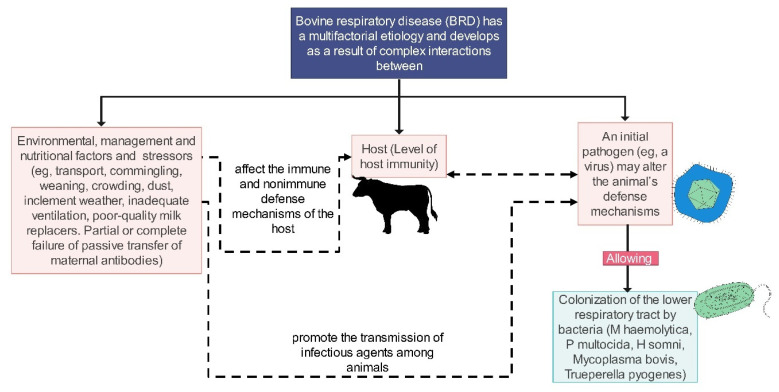
Diagramtic illustration displaying the interplay between environment, host and BRD pathogens.

Prior research has established the use of both cohort and individual animal characteristics upon arrival, along with individual treatment records, to identify risk factors for the development of BRD [58,59]. This approach has led to the creation of predictive prognostic tools that can assess the risk for individual animals at the onset of their first treatment [266,267]. Extensive efforts have been directed toward understanding the complex and polymicrobial nature of BRD by analyzing host factors, therapeutic interventions, etiological agents, and environmental stressors in connection with the disease’s progression (Figure 2) [12,268,269]. Recent studies have focused on predicting susceptibility to BRD and its long-term outcomes [202,270]. Despite these advancements, the reliability of clinical analyses and predictive models remains controversial, and a comprehensive understanding of the host-pathogen dynamics and the pathogenesis of BRD is still incomplete.

A recent study found a positive association between post-viral infection increases in glucose levels and extended survival rates (*p* < 0.05) [26]. In contrast, initial lactate concentrations at the time of BRD diagnosis did not directly predict mortality in natural cases. However, an incremental increase in lactate levels by 1-log (measured on days 3, 6, 9, and 15 following initial treatment) significantly heightened the mortality risk by a factor of 36.5 (95% CI: 3.5–381.6) [26]. Further research is warranted to ascertain the precise prognostic significance of lactate measurements. Additionally, studies on pathogen detection have revealed that calves testing positive for *M. bovis* via nasal swab were at an increased risk of death following their initial BRD treatment (odds ratio [OR], 3.0), as well as at a higher likelihood of requiring second (OR, 3.3) or third (OR, 3.2) treatments [271].

## 8. Conclusions

Herein, this article presents a comprehensive review of the diagnostic methods currently used to identify BRD. While existing diagnostic techniques such as animal imaging and behavioral analysis provide valuable insight, they often suffer from observer subjectivity and prohibitive costs.

Future diagnostics should strive to automate processes and minimize subjectivity by leveraging quantitative analysis. Efforts have already been made in this direction, with the development of monitoring systems that track individual animal movements over time. Moreover, preliminary models that use data from these systems to identify animals at higher risk for BRD show promise. Further advancements should prioritize early detection, enabling prompt therapeutic intervention. To enhance these models, additional metrics like temperature variations, respiration rates, effort, and the presence of nasal discharge should be monitored autonomously.

Finally, in terms of imaging technology, advancements should aim to deploy high-quality, autonomous imaging in the field. Such technology could categorize and prioritize animals based on the severity of BRD symptoms, allowing for treatment plans tailored to the disease’s progression and potentially improving recovery rates.

In general, future diagnostics should collect data that is quantitative, autonomous, continuous, and field-appropriate. This approach promises more timely and informed therapeutic interventions, thereby improving recovery prospects. However, it is crucial to recognize that no single diagnostic technology will suffice on its own; a multifaceted approach that enhances traditional diagnostics will likely yield the best clinical outcomes.

## Figures and Tables

**Table 2:** Some available Biomarkers for BRD Diagnosis and Prognosis. Abbreviations in the table include PCR for Polymerase Chain Reaction, TIBC for Total Iron Binding Capacity, TS for Transferrin Saturation, Fe for Iron, and DE for Differentially Expressed. BRD refers to Bovine Respiratory Disease, Hp to Haptoglobin, TUS to Thoracic Ultrasound, IL-8 to Interleukin-8, NP to Neopterin, and IF-γ to Interferon-gamma. BCV is Bovine Coronavirus, Acc is Accuracy, VD is Visual Diagnosis, TD is Temperature Diagnosis, LAD is Lung Auscultation Diagnosis, and LLD is Lung Lesion Diagnosis, NMR is Nuclear Magnetic Resonance, BVDV is Bovine Viral Diarrhea Virus, LDL is Low-Density Lipoprotein, LDH is Lactate Dehydrogenase, cTnI is Cardiac Troponin I, WBC is White Blood Cell, APPs are Acute Phase Proteins, BHV-1 is Bovine Herpesvirus 1, and *M. haemolytica* is *Mannheimia haemolytica.* VCD stands for Visual+Clinical Diagnosis, SAA for Serum Amyloid A, LPO for Lipid Peroxidation, Alb for Albumin, R-GSH for Reduced Glutathione, SOD for Superoxide Dismutase, CAT for Catalase, RNA for Ribonucleic Acid. Specific biomarkers include IFIT3 for Interferon Induced Protein with Tetratricopeptide Repeats 3, IFI6 for Interferon Alpha Inducible Protein 6, MX1 for MX Dynamin Like GTPase 1, ISG15 for Ubiquitin Like Modifier ISG15, and OAS2 for 5′-Oligoadenylate Synthetase 2. mRNA refers to Messenger Ribonucleic Acid. Genes like CFB (Complement Factor B), MARCO (Macrophage Receptor with Collagenous Structure), ALOX15 (Arachidonate 15-Lipoxygenase), MCF2L (MCF.2 Cell Line Derived Transforming Sequence-Like Protein), and SLC18A2 (Solute Carrier Family 18 Member A2) are also included.

Sample Size Included in the Study	Diagnostic Methods	Key Findings	Reference
25 BRD, 10 healthy	Clinical signs, PCR	Low TIBC, TS, serum Fe; high ferritin in infected animals	[201]
12 BRSV challenged, 6 controls	Transcriptomic analysis	281 DE genes with fold change >2	[192]
162 healthy beef calves	Clinical signs, gel electrophoresis, mass spectrometry	Lowered proteins post transportation/weaning (lowered annexins A1, A2, calcyphosin, peroxiredoxin I, macrophage capping protein, superoxide dismutase and dihydrodiol dehydrogenase 3)	[223]
477 preweaned dairy calves	TUS, auscultation	Lower Hp in controls; 82% specificity for BRD	[34]
69 BRD infected, 20 healthy	Visual, clinical exams	Predictive biomarkers for BRD treatment response (PCT, IL-8, NP, IF-γ, Hp and IL-1β)	[218]
Not specified	In silico tools	Potential BCV diagnostic markers identified (AREB6, YY1, NKX2, and LMO2)	[224]
297 (149 BRD, 148 non-BRD)	Multiple diagnostics; metabolomics assessment	Blood metabolomics accuracy: varying Acc rates for methods (Acc = 0.85) compared to VD, However, (Acc = 0.65) to TD, Compared to LAD score (Acc = 0.61), and LLD (Acc = 0.71).	[169]
50 BRD, 10 healthy	NMR metabolomics analysis	Metabolite alterations in diseased calves (Alterations in metabolites, increases in phenylalanine, 2-methyl glutarate, phosphatidylcholine, and decreases in dimethylsulfone, ethanol, acetate, propionate, free cholesterol, allantoin, cholesterol (–C18))	[201]
Four male Holstein calves control and five challenged)	microRNA analysis	Potential BVDV exposure biomarkers identified (Bta-miR-423-5p or bta-miR-151-3p)	[225]
200 samples (100 serum, 100 nasal swabs)	Volatile organic compounds analysis	Different compounds in ‘normal’ vs. ‘sick’ cattle samples; Four for nasal swabs, while five differed in their serum samples, with phenol being the most common	[33]
54 weaned calves (Healthy, moderate, severe BRD)	LDH activities, serum cTnI concentrations, clinical index scores	Severe BRD calves exhibited elevated LDH activities, serum cTnI concentrations, neutrophilia, lymphopenia, and increased WBC counts. Clinical index scores determined disease severity.	[33]
20 animals	Proteomic and metabonomic studies; viral challenge model	Proteomic studies identified APPs (HP and apolipoprotein AI) linked to viral respiratory infections. Metabonomics and elemental analyses detected biomarkers for disease outcome (glucose and lactate) and viral infection (LDL, glucose, phosphorus, iron, and valine). Significant serum proteome changes occurred on day 4 postviral infection compared to preinfection (day 0) samples, after BHV-1 infection challenge and *M. haemolytica*.	[26]
300 steers (148 with BRD, 152 without)	Metabolomics analysis; various diagnostic methods compared	VD, VCD, and LLD were diagnostic methods for BRD. In the validation dataset, metabolomics showed high accuracy in detecting BRD using VD (Acc = 0.85) and VCD (Acc = 0.81), but lower accuracy using LLD (Acc = 0.74).	[226]
26 induced BRD	Clinical signs, biomarker analysis for lung lesions	Multiple biomarkers and clinical signs analysis post-experimental infection.	[227]
482 heifers	Biomarker analysis (HP, SAA, LPO, Alb, R-GSH, SOD, and CAT)	Oxidative stress association with APPs in calves with naturally occurring BRD.	[228]
43 animals (25 with BRD, 18 without)	RNA sequencing of whole blood	Distinct immune response profile in animals with BRD; potential diagnostic use of blood transcriptome. In infection and inflammation DE genes up, and maintenance genes and metabolic and cell growth down.	[191]
864 blood samples (300 Entry, 466 Pulled, 98 Close-out)	Biomarker identification at different stages	Prediction of sick cattle at entry by specific biomarkers including IFIT3, IFI6, MX1, ISG15, and OAS2.	[190]
48 cattle within first 28 days of arrival (24 with BRD, 24 healthy)	mRNA sequencing; stratified by antimicrobial use	Biomarkers to predict BRD severity and occurrence; gene expression associated with disease progression and severity differentiation. Six genes (CFB, MARCO, ALOX15, MCF2L, SLC18A2, LOC100335828) predict BRD severity and development from arrival blood samples.These genes fall into two groups: Treated_1 vs. healthy/treated_2+: Increased neutrophil activation, antimicrobials, and keratinization. Treated_2+ vs. healthy/treated_1: Decreased leukocyte activity, increased nitric oxide and alternative complement.	[195]

## Data Availability

No new data were created or analyzed in this study. Data sharing is not applicable to this article.
